# Crystal and Molecular Structure of Novel Silver-Diphosphine Complexes

**DOI:** 10.1155/MBD.1994.526

**Published:** 1994

**Authors:** Francesco Caruso, Luigi M. Venanzi

**Affiliations:** 1 Instituto di Strutturistica Chimica CNR, Box 10 Monterotondo Stazione Roma I - 00016 Italy; 2 Eidgenossische Technische Hochschule Universitätstraße 6 Zürich CH - 8092 Switzerland


					OF
CRYSTAL AND MOLECULAR STRUCTURE
NOVEL SILVER-DIPHOSPHINE COMPLEXES
Francesco Caruso and Luigi M. Venanzi2
Instituto di Strutturistica Chimica, CNR, Box 1 O, 00016 Monterotondo Stazione, Roma, Italy
2 Eidgenossische Technische Hochschule, Universit&tstral3e 6, CH 8092 ZOrich, Switzerland
It has been shown that the bis-chelated complex Au(dppe)2CI, dppe diphenylphosphinoethane
and derivatives are active against a series of tumor cell lines [1]. Later, closely related silver
diphosphine complexes were shown to be cytotoxic against B melanoma cells in vitro and P 388
leukemia in mice [2]. Furthermore, Cu-diphosphine complexes are also biologically active [3]. The
diphosphine ligands have been suggested as the cytotoxic species, since they are also active but
less than some of their metal complexes [3]. Chelation seems to be an essential condition for
biological activity in these complexes.
We have been studying the coordination properties of silver-phosphine complexes ([4] and
references therein) and will review this chemistry, focusing on chelation conditions. We describe
some Ag-complexes of the ligands 1,2-bis(diphenylphosphinomethyl)benzene and 1,3-bis(diphenyl-
phosphinomethyl)benzene. The former ligand is able to give a new type of silver chelate species.
References
[1] C.K. Mirabelli, D. T. Hill, L. F. Faucette, F. L. McCabe, G. R. Girard, D. B. Bryan, B. M.
Sutton, J. O'Leary Bartus, S. T. Crooke, and R. K. Johnson, J. Med. Chem. 1987, 30, 2181-
2189.
[2] S.J. Berners-Price, R. K. Johnson, A. J. Giovenella, L. F. Faucette, C. K. Mirabelli, P. J.
Sadler, J. Inorgan. Biochem. 1988, 33, 285-295.
[3] R.M. Snyder, C. K. Mirabelli, R. K. Johnson, C. Sung, L. F. Faucette, F. L. McCabe, J. P.
Zimmerman, M. Whitman, J. C. Hempel, and S. T. Crooke, Cancer Res. 1986, 46, 5054-
5060.
[4] M. Camalli, F. Caruso, S. Chaloupka, and L. M. Venazi, Helv. Chim. Acta, 1988, 71, 703-711.
526

				

